# ﻿Mitochondrial genome and transcription of *Shiraia*-like species reveal evolutionary aspects in protein-coding genes

**DOI:** 10.3897/imafungus.16.138572

**Published:** 2025-02-20

**Authors:** Xiao-Ye Shen, Xue-Ting Cao, Xiao-Bo Huang, Lan Zhuo, Hui-Meng Yang, Li Fan, Cheng-Lin Hou

**Affiliations:** 1 College of Life Science, Capital Normal University, Beijing, Xisanhuanbeilu 105, Haidian, Beijing100048, China Capital Normal University Beijing China

**Keywords:** *atp6*, comparative analysis, gene re-arrangement, hypocrellin, mitogenome, *Pseudoshiraiaconidialis*, *Shiraia*

## Abstract

*Shiraia*-related species are well-known bambusicolous fungi in *Dothideomycetes* class, with high value in traditional medicine for producing hypocrellin, as an anticipated photosensitiser. The complete mitogenomes of hypocrellin-producing *Pseudoshiraiaconidialis* strains were analysed in the present study, with functional gene variations through comparative genomics and transcriptomics. Five strains (ZZZ816, CNUCC1353PR, JAP103846, CNUCC C72, CNUCC C151) were sequenced, which indicated similar genome characteristics. Two of them possess an extra *atp6* gene, and the associated variable fragment “*HSP1-HSP2-atp6_2*” correlates closely with hypocrellin production capacity. Therefore, these five strains were divided into three groups: ZZZ816 and CNUCC1353PR possessing high production efficiency, CNUCC C72 and JAP103846 with low yield and CNUCC C151 as a transition type. The gene expression changes were screened under various conditions. ZZZ816-related species showed significant changes in mitochondrial genes, especially *HSP1*, *HSP2* and *atp6_2*, linked closely to hypocrellin synthesis and stress response; *rps3* expression also consistently correlated with hypocrellin production. JAP103846 group showed a stable expression pattern divergently, except for *rps3* suppression by blue light. These findings would provide new insights into secondary metabolite regulation and ROS resistance.

Above all, this study conducted the comprehensive analysis of *Shiraia*-like fungi mitogenomes and functional gene expression, which can update the understanding of fungal evolution and potential for improved hypocrellin production.

## ﻿Introduction

Species associated with *Shiraia* are well-known as medicinal macrofungi in ethnopharmacology and for specifically inhabiting bamboo plants. Extracts from their fruit bodies and mycelia are commonly used in traditional healing to alleviate inflammation, apoplexy and sciatica. Recent studies have reported an increasing number of natural bioactive products from these strains, of which hypocrellin and the similar photodynamic pigments were the most fascinating agents. These kinds of compounds, all belonging to perylenequinone, are widely used in the pharmaceutical, food and agricultural industries (Khiralla et al. 2022).

*Shiraia* spp., as typical *Dothideomycetes* fungi, have been found on various tissues of bamboos, including branches, leaves and seeds. Phylogenetic analyses revealed that almost all of these strains can be traced back to *Shiraiabambusicola* P. or located in the neighbouring groups (Liu et al. 2013). Interestingly, the industrial strains currently used in hypocrellin production, were not actually identified as *S.bambusicola*. Instead, the majority of these isolates were in the lineages sister to *Shiraia*, while a few others were dispersed amongst more distant groups (Tong et al. 2021). Although the species name “*S.bambusicola*” has been used for a long time for the strains with high yield of hypocrellin in the fermentation industry, a monotypic genus *Pseudoshiraia* was established to accommodate *P.conidialis*, which was considered as the real name for the industrial strains (Tong et al. 2021).

Mitochondria are organelles responsible for oxidative metabolism and which play a crucial role in cell functioning. They have their own genomes, which contain the genes encoding functional proteins, ribosomes and transferring nucleic acids. These genes are important for biological functions, growth, development and stress response in eukaryotes and also in immune system regulation and plant cytoplasmic male sterility (Chen et al. 2017; Andrieux et al. 2021). Recent reports have shown that the changes in the mitochondrial genome play a significant role in the growth and development, oxidative stress and environmental responses of eukaryotes (Cai et al. 2021; Guan et al. 2021; Du et al. 2022). In animals and humans, mutations from specific mitochondrial genome genes or even bases are closely linked to important diseases (Du et al. 2022; Pickett et al. 2022; Zhong et al. 2022). Moreover, the development of mitochondrial genomics has provided substantial support for the analysis of animal systematics and population genetics research (Zhang et al. 2021; Garcia-Souto et al. 2022; Wolfsberger et al. 2022). However, studies on fungal mitochondrial genomes are still scarce, particularly for the relationship between functional gene variation and mycelial growth, development and stress response.

Most fungal mitochondrial genomes are circular, with a few being linear. They are usually rich in AT and have a large range of sizes, ranging from 1.1 kb (*Spizellomycespunctatus* (W.J. Koch) D.J.S. Barr) to 272 kb (*Morchellaimportuna* M. Kuo, O’Donnell & T.J. Volk; Forget et al. (2002); Liu et al. (2020)). Variation in size is mainly influenced by differences in auxiliary genes, such as RNA and DNA polymerases, reverse transcriptases and transposases, variation in intergenic regions and the presence/absence of introns. Even in close species, some fungal mitogenomes also exhibit significant differences in gene arrangement, repeat sequence content, gene number and intron type (Li et al. 2020; Araujo et al. 2021; Fonseca et al. 2021; Li et al. 2021). However, core gene numbers and function often remain relatively stable. The corresponding functions typically cover 11 representative genes encoding respiratory chain subunits (*cox1*, *cox2*, *cox3*, *cob*, *nad1-6* and *nad4L*), three ATP family genes encoding ATP synthase complex subunits (*atp6*, *atp8* and *atp9*), as well as genes encoding rRNA (*rns* and *rnl*), tRNA (*trn*) and *rps3*, which participates in mitochondrial ribosome assembly (Paquin et al. 1997; Kouvelis et al. 2004). There are only a few cases with significant changes in these genes. For example, certain fungal species display *rps3* deletion or transfer to the nuclear genome (Korovesi et al. 2018) and *nad* family gene deletion appears in the mitogenome of the subphylum *Saccharomycotina* O.E. Erikss. & Winka and the family *Saccharomycetaceae* Luerss (Christinaki et al. 2022).

Hypocrellin is lauded as the next generation of photosensitier and their photodynamic effect is closely related to the synthesis of reactive oxygen species (ROS) and mitochondrial apoptosis (Qi et al. 2019; Niu et al. 2020). When hypocrellin is irradiated with visible light, it reacts with cell substrates in a triple state, producing free radicals. These free radicals then transfer energy to oxygen, generate ROS, which plays a crucial role in antibiotic activity (Zhang et al. 1998; Lu et al. 2006). Moreover, ROS can also react with the cytoplasmic membrane, disrupting the transport system and enzyme activity, as reported by Hamblin et al. (Hamblin and Hasan 2004). Recent studies have shown that hypocrellin significantly increases ROS generation and damages the cell membrane of *Candidaalbicans*. Hypocrellin-mediated antimicrobial photodynamic therapy (aPDT) alters the structure of the cell wall and membrane, facilitating the transportation of hypocrellin into the cell. Hypocrellin can also increase the calcium ion concentration in the cytoplasm and mitochondria of *Candidaalbicans*, possibly through the transfer of cytoplasmic calcium (Yang et al. 2019). The increase in mitochondrial calcium level is able to trigger the release of pro-apoptotic proteins, leading to the aging and death of *Candidaalbicans* (Carraro and Bernardi 2016). Qi et al. (2019) suggested that the opening of PT pores induced by ROS was the most critical step in this process, causing significant swelling of mitochondria and damage to respiratory function.

As mentioned above, hypocrellin has the ability to directly interfere with mitochondrial function and its amount inside cells ought to determine the actual biological activity. Therefore, it is interesting to know how these unmodified wild-types with high-yield coordinate the negative effect of hypocrellin *in vivo* and normal growth of mycelia. In the *Shiraia*-like species, strains with varying production capabilities displayed distinct structural sequences and expression levels of their mitogenomes. The analyses of these mitochondrial genomes would not only reveal evolutionary relationship between the fungal species, but also assist to identify superior production strains, potentially improving the synthesis efficiency of hypocrellin and related natural products.

## ﻿Materials and methods

### ﻿Sample information

The strain of *Shiraia*-like species, CNUCC1353PR, was isolated from bamboo plants in the Pu’er District, Yunnan, ZZZ816 from bamboo seeds in Guangxi, endophytic fungi CNUCC C151 and C72 from *Bambusaemeiensis*. The strain JAP103846 was received from *Phyllostictabambusae* in Japan. All strains have been identified based on both morphology and multigene phylogeny (Tong et al. 2021). The strains CNUCC 1353PR and ZZZ816 are preserved in the China Forestry Culture Collection Center (CFCC), CNUCC C72 and C151 in the Capital Normal University Culture Collection Center (CNUCC) and JAP103846 in the Biological Resource Center, NITE (NBRC), Japan.

The fungal strains were cultured separately on 2% potato dextrose agar media (PDA), which contains 200 g/l potato, 20 g/l dextrose and 20 g/l agar at pH 6.0. The mycelia from subcultured colonies were scraped from the surface of the agar and frozen in liquid nitrogen for genomic DNA extraction. The extraction process followed the instructions of the DNeasy Fungal Mini Kit (Qiagen) and the modification of Shen et al. (2014) and the interference of nuclear DNA was tested by PCR for the target regions of ITS rDNA (Tong et al. 2021). The extracted DNA was stored at −80 °C for future use.

### ﻿Mitogenome annotation and sequence analysis

Total DNA was randomly sheared into fragments of approximately 350 bp. These fragments were then sequenced on an Illumina NovaSeq platform in 2 × 150 bp reads at Novogene Co. Ltd. (Tianjin, China). The sequencing data underwent quality filtering. Paired reads were discarded if more than 10% of bases were uncertain in either read or if the proportion of low-quality bases (Phred quality ≤ 20) exceeded 40% in either read. To ensure the accurate mitochondrial genome, the obtained sequencing reads were de novo assembled using two independent programmes: SPAdes (Prjibelski et al. 2020) and GetOrganelle (Jin et al. 2020). Primers were then designed, based on the obtained mtDNA fragments for PCR and sequencing, resulting in the successful assembly of the complete mtDNA of *Shiraia*-like species.

For the annotation of protein-coding genes, the obtained sequences were compared with the GeneBank database using BLASTx. The “Mold Mitochondrial” parameter setting was used for codons and default settings were used for other parameters. Additional annotation analysis was performed using software tools such as PGA (Qu et al. 2019), Mitofinder (Allio et al. 2020), MITOS Web Server (Bernt et al. 2013) and Geseq (Tillich et al. 2017), along with necessary manual corrections.

The same annotation method used for protein-coding genes was applied to annotate rRNA in the mitochondrial genome, ensuring accurate identification and annotation of the rRNA genes. For the annotation of tRNA, the software tRNAscan-SE was utilised, specifically designed for the identification of tRNA genes with the secondary structure in mitochondrial genomes (http://lowelab.ucsc.edu/tRNAscan-SE/). Open reading frames (ORFs) within introns and intergenic regions were identified using ORF Finder (https://www.ncbi.nlm.nih.gov/orffinder/), considering only ORFs ≥ 300 bp.

To investigate the protein-coding genes in the mitogenome of *Shiraia*-like species, we conducted codon-usage analysis using the DAMBE software (Data Analysis and Molecular Biology and Evolution) (Xia 2018). This software was used to assess the relative synonymous codon usage (RSCU) of these genes. Furthermore, we computed the AT and GC skew of the mitochondrial genome using the formula proposed by Perna and Kocher (Perna and Kocher 1995). The AT skew measures the asymmetry between adenine (A) and thymine (T) nucleotides, while the GC skew quantifies the asymmetry between guanine (G) and cytosine (C) nucleotides. These skew calculations provided insights into the nucleotide bias within the mitogenome.

In other word, the RepeatMasker software was utilised to analyse the repetitive sequences in the mitochondrial genome (Saha et al. 2008). The analysis was performed with the parameter setting “DNAsource:fugu”, which accounted for the specific characteristics of the *Shiraia*-like species. Default settings were used for other parameters to ensure a comprehensive analysis of repetitive sequences.

### ﻿Transcriptional analysis of functional genes

To analyse the transcription of mitochondrial genes and validate the annotations, RNA-Seq was performed on mycelia cultivated on PDA plates. Total RNA was used as the starting material for library construction. The process involved enriching mRNA with a polyA tail using Oligo (dT) beads, fragmenting the mRNA randomly with divalent cations, synthesising the first strand cDNA with M-MuLV reverse transcriptase, degrading the RNA chain with RNaseH, synthesising the second strand cDNA, purifying the double-stranded cDNA, A-tailing it and ligating it with sequencing adapters. A 370-420 bp cDNA fragment was selected and PCR amplified to create the final library. The library was then sequenced using Illumina NovaSeq platform (2 × 150 bp reads) at Novogene Co. Ltd. (Tianjin, China) after passing quality control.

The raw sequence data obtained from sequencing need to be filtered to obtain clean data for subsequent analysis. This involves removing reads with undetermined nucleotide information (reads with N), reads with adapters and low-quality reads (reads with more than 50% bases having a Qphred score ≤ 20). Then, the clean reads of the paired-end data were aligned to the reference genome using STAR software (Dobin et al. 2013). The expression of mitochondrial genes was quantified using featureCounts from Subread, which normalised the raw read counts by correcting for sequencing depth (Liao et al. 2014). Differential gene expression analysis was performed using three different statistical methods (edgeR, limma and DESeq2) for samples with biological replicates (Liu et al. 2021). The p-value for hypothesis testing was calculated and multiple hypothesis testing correction was applied to obtain the false discovery rate (FDR) values (commonly represented as padj).

### ﻿Phylogenetic analysis of *Shiraia*-like species

To determine the phylogenetic position of *Shiraia*-like species in the class *Dothideomycetes*, the nucleic acid and amino acid sequences of the mitochondrial genomes of the target fungi were downloaded from the NCBI database and FUNGAL databases. The relevant analysis was performed using the alignments of the 14 protein-coding genes (PCGs) of complete or near-complete mitogenomes of the *Pleosporales* and related taxa, including *atp6*, *atp8*, *atp9*, nad1, *nad2*, *nad3*, *nad4*, nad5, *nad6*, *nad4L*, *cob*, *cox1*, *cox2* and *cox3*. Amino acid sequences were aligned using MAFFT (https://www.ebi.ac.uk/jdispatcher/msa/mafft) and the non-well-conserved regions were then improved manually by MEGA7 (Kumar et al. 2016). *Ogataeaangusta* (Teun., H.H. Hall & Wick.) S.O. Suh & J.J. Zhou and *Pichiapastoris* (Guillierm.) Phaff were selected as outgroups.

A Python script was used to concatenate the different protein amino-acid sequences of the same species and convert them to the NEX format required by the MrBayes software. The outgroups were set as the yeast group. Maximum Likelihood (ML) analysis was performed with IQ-TREE 2.2.0 (Minh et al. 2020) and the best-fit model for *atp6* is PMB+G4, for *atp8* mtZOA+I+G4, for *atp9* cpREV+I+G4, for *cob* is PMB+G4, for *cox1* PMB+G4, for *cox2* PMB+F+G4, for *cox3* is PMB+G4, for *nad1* WAG+F+G4, for *nad2* VT+F+G4, for *nad3* is PMB+G4, for *nad4* WAG+F+G4, for *nad4L* PMB+G4, for *nad5* PMB+F+G4 and for *nad6* VT+F+G4. ML bootstrap replicates (1000) were computed in IQ-TREE using a rapid bootstrap analysis and search for the best-scoring ML tree. The Bayesian analysis was performed with MrBayes 3.1.2 (Huelsenbeck et al. 2001; Ronquist et al. 2003). The analysis of four chains were conducted for 100 000 000 generations with the default settings and sampled every 1000 generations, halting the analysis at an average standard deviation of split frequencies of 0.01. The first 25% of the trees were removed as burn-in. Nodes were considered strongly supported if they received a posterior probability ≥ 0.95 (for BI) or bootstrap values ≥ 70% (for ML).

### ﻿Evolutionary rates of the mitochondrial genes

In addition to *Shiraia*-like species, this study also included other associated species with hyporellin, which had available mitogenomes. To understand the relationship between mitogenome evolution and the presence of hypocrellin, we compared these mitogenomes, based on genome size, gene content, gene order and introns. Evolutionary rates are commonly calculated using synonymous sites (dS), non-synonymous substitution sites (dN) and their ratios (dN/dS). To measure these values, we focused on 12 protein-coding genes (as shown in the “Phylogenetic Analysis of Shiraia-like Species” section above). The values of Ka, Ks and Ka/Ks were calculated by concatenating these genes using MEGA 7.0 with codons as parameters (Gap opening penalty: 400; Gap extension penalty: 0.2; Delay divergent cutoff: 30%). The dN/dS ratio was determined using DnaSP ver. 524.

### ﻿Availability of data

The mitogenome sequence of *Shiraia*-like species has been deposited in the Genome Sequence Archive (GSA) at the National Genomics Data Center (NGDC) under accession numbers C_AA085157, C_AA085158, C_AA085159 and C_AA085160, except ZZZ816, which was revised and modified from our previous data (accession number KM382246). RNA-Seq data have been downloaded from the NCBI Sequence Read Archive (SRA) database with the BioProject accession numbers PRJNA323638, PRJNA475310, PRJNA477419 and PRJNA544773.

Standard hypocrellin was purchased from Sichuan Weiqi Biotechnology Co. Limited (Chengdu, China), while the test array was inferred from our previous article (Tong et al. 2021) and modified accordingly.

## ﻿Results and discussion

### ﻿Assembly and organisation of the mitogenome

In this study, five representative strains of *Shiraia*-like species were singled out with different habitats and host plants, i.e. CNUCC1353PR (isolated from Pu’er, Yunnan), ZZZ816 (from bamboo seeds in Guangxi), CNUCC C151 and CNUCC C72 (from *Bambusaemeiensis*) and JAP103846 (from *Phyllostictabambusae* in Japan). The strain screening relied on different morphological characteristics and the presence of divergent natural products. The strains CNUCC1353PR and ZZZ816 showed significantly higher efficiency in the synthesis of hypocrellin, while the fermented production of CNUCC C151 was relatively unstable. The strains CNUCC C72 and JAP103846 did not show the presence of relevant active ingredients (Suppl. material 1).

**Figure 1.**
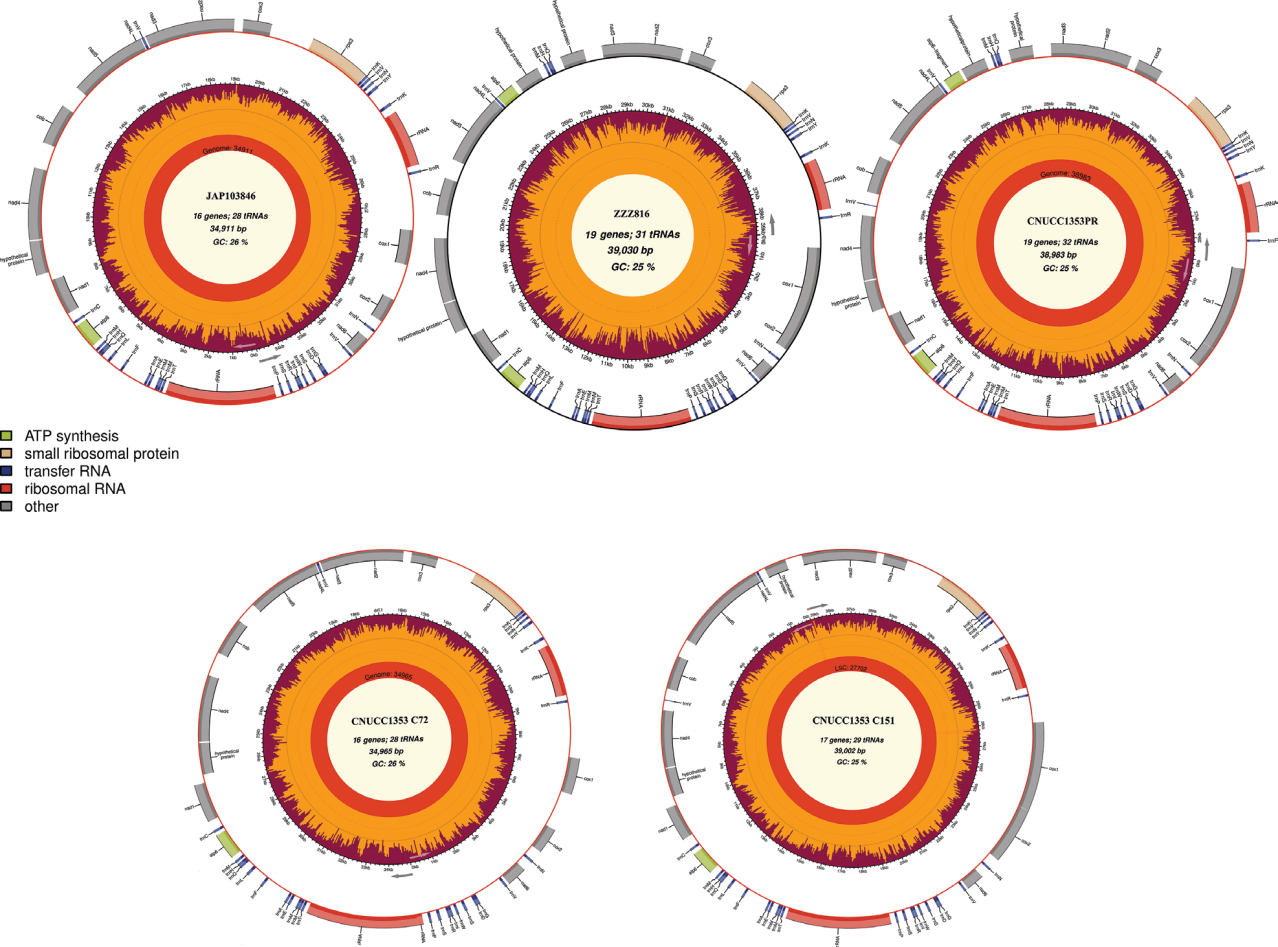
The circle maps of the complete mitochondrial genomes of *Shiraia*-like fungi (*Pseudoshiraiaconidialis*), covering strains JAP103846, ZZZ816, CNUCC1353PR, CNUCC C72 and CNUCC C151.

**Figure 2.**
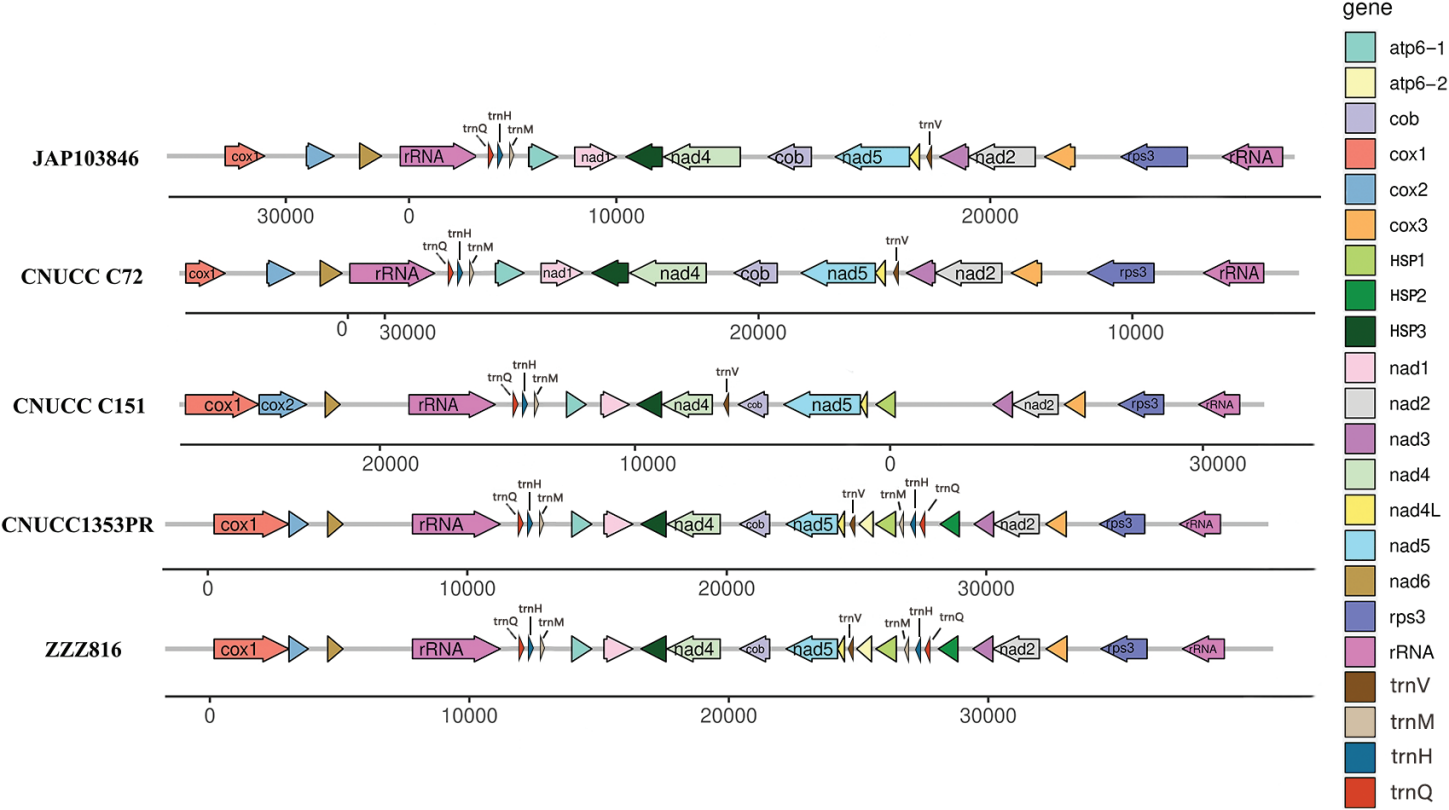
Gene order comparison of protein-coding genes and rRNAs from the entire mitochondrial genomes of five *Shiraia*-like species.

The five complete mitogenomes of the aforementioned species were assembled, modified and finalised separately, using high-throughput sequencing data and PCR cloning verification (Fig. 1). The analyses of conserved genes between these sequences and other corresponding sequences revealed that these strains could be directly divided into two major groups, representative of all species which have been applied in fermentation production (Tong et al. 2021). As shown in Table 1, the finished mitochondrial genomes of *Shiraia*-like species range in size from 34.911 kb to 39.030 kb, with relatively small divergence in size. These mitogenomes all contain 12 protein-coding genes (PCGs), including ATPase subunits 6 (*atp6*), cytochrome oxidase subunits 1 to 3 (*cox1-cox3*), cytochrome b (*cob*), NADH dehydrogenase subunits 1–6 and 4 L (*nad1-6* and *nad4L*) and two rRNAs (small subunit ribosomal RNA/rns and large subunit ribosomal RNA/rnl) (Fig. 2). Additionally, the *atp8* and *atp9* genes, normally present in fungal mitochondria, have been transferred to the nuclear genome. It is worth noting that a homologous gene of *atp6* appears in ZZZ816 and CNUCC1353PR and the sequences between the twin copies are almost the same, except for the head and tail. The GC content of the five mitogenomes ranges from 25.19% to 25.57%, with an average GC content of 25.36%. JAP103846 has the highest GC content, while ZZZ816 has the lowest. Two of the GC skews, CNUCC C72 and CNUCC C151, are positive, while the others are negative. Most of the AT skews are negative, except for CNUCC1353PR (Table 2).

**Table 1.** Annotation information of the mitochondrial genomes of *Shiraia*-like species.

Genomes features	CNUCC C72	JAP103846	CNUCC C151	CNUCC 1353PR	ZZZ816
Genomes size (bp)	34,965	38,983	39,002	34,911	39,030
G+C content (%)	25.55	25.57	25.28	25.21	25.19
No. of protein-coding genes	14	14	15	17	17
G+C content of protein-coding genes (%)	26.57	26.62	27.47	27.28	27.1
Structural proteins coding exons (%)	45.32	45.41	43.82	47.48	47.33
No. of rRNAs/tRNAs	2/28	2/28	2/29	2/32	2/32
G+C content of RNA genes (%)	35.44	35.44	35.41	35.53	35.4
rRNAs+tRNAs (%)	20.21	20.24	18.20	18.77	18.92
Coding regions (%)	65.53	65.65	62.02	66.25	68.96
Intergenic regions (%)	34.47	34.35	37.98	33.75	30.51
No. of introns	0	0	3	1	1
No. of intronic ORFs	0	0	0	0	1
Introns (%)	0	0	8.714	3.245	3.24

**Table 2.** The base content of mitochondrial genomes.

Strain	A%	T%	C%	G%	GC%	AT%	GC-skew	AT-skew
CNUCC C72	37.23	37.23	12.83	12.71	25.55	74.45	-0.00470	-0.00038
JAP103846	37.21	37.22	12.72	12.84	25.57	74.43	0.004818	-0.00015
CNUCC C151	37.28	37.44	12.67	12.60	25.28	74.72	-0.002840	-0.00213
CNUCC1353PR	37.41	37.39	12.58	12.62	25.21	74.79	0.001425	0.000240
ZZZ816	37.41	37.40	12.57	12.62	25.19	74.81	0.001027	-0.00072

### ﻿Surveys of conserved PCGs in mitogenomes

A series of PCGs in the mitochondrial genomes typically exhibits relative conservation. Sequence alignment and similarity analysis have shown that the base variation and arrangement of the mitogenome remain relatively stable. However, the comprehensive and in-depth comparison reveals some noticeable differences, particularly in the insertion of exogenous fragments or the loss of the original sequence. These differences may have a significant impact on protein function.

One notable difference is the fragment between position *nad4L* and position *nad3*. This fragment showcases the diversity of mitogenomic evolution, as different strains contain different parts of the DNA regions (Suppl. material 2). Amongst the strains, ZZZ816 and CNUCC1353PR have the longest length of the relevant fragment. Within this fragment, *atp6-2*, *HSP1* and *HSP2* are arranged in a specific order. On the other hand, JAP103846 and CNUCC C72 have lost most of this region. CNUCC C151 partially contains the fragment, with only *HSP2* remaining in the sequence. As the longer mitogenome, CNUCC C151 is suggested to represent the middle evolutionary route and has eliminated two specialised proteins. Based on the change in the *HSP1_HSP2_atp6-2* fragment, these strains can be divided into three groups, which interestingly correspond to the synthetic efficiency of hypocrellin.

### ﻿Intron dynamics amongst the Shiraia–like species

In the fungal kingdom, the mitogenomes of *Shiraia* spp. are characterised as the small size, mainly due to the low number of introns (Table 1). In the existing sequences, the intron of *cox1* is highly conserved, with homologous sequences found in the same position from closely-related taxa of *Dothideomycetes*, such as *Exserohilumrostratum* and *Bipolariscookei*. Strain CNUCC C151 has two extra introns in *cox2* and *nad5*, containing GIY-YIG nuclease domain and LAGLIDADG endonuclease, respectively, which are not observed in the close species. Notably, these two introns have few homologous sequences in *Dothideomycetes*, but many similar sequences are found in the adjacent class of *Sordariomycetes*. For instance, *Epichloe* in *Hypocreales* has a high similarity with both introns (Treindl et al. 2021). In-depth comparison of the *HSP1_HSP2_atp6-2* fragment revealed that CNUCC C151 was located between the ZZZ816 and CNUCC1353PR group and the JAP10346 and CNUCC C72 group. However, from the perspective of introns, these taxa are likely to have more complex evolutionary trajectory.

### ﻿rRNA and tRNA genes

The five mitochondrial genomes all contain two rRNA genes, *rns* and *rnl* and between 27 to 31 tRNAs, which encode 20 standard amino acids. Despite variations across different strains, the type and order of tRNAs generally remain stable. Separately, strains JAP10346 and CNUCC C72 consistently have 28 tRNAs (Suppl. material 6: tables S1, S2); strains ZZZ816 and CNUCC1353PR have an increased total of 31 tRNAs (Suppl. material 6: tables S3, S4); CNUCC C151 has the fewest tRNAs, with only 27, but it additionally contains two incomplete *trn*V fragments (Suppl. material 6: table S5). One of these incomplete *trn*V fragments is complete in strains ZZZ816 and CNUCC1353PR, while CNUCC C151 has lost part of the sequence at the same location. JAP10346 and CNUCC C72 have lost even more of this fragment, as it cannot be found directly through sequence analysis. Another incomplete fragment is present in strains ZZZ816, CNUCC1353PR and CNUCC C151, but it is hidden in the non-coding sequence of JAP10346 and CNUCC C72 due to a single-nucleotide variant (SNV). The sequence variations in these fragments may correspond to the emergence patterns of *HSP* genes. The former fragment is notably adjacent to *HSP1* of CNUCC C151 and its absence appears closely related to variations in this region, particularly in the function of *HSP1* and *HSP2*. As tRNA mutations commonly affect mitochondrial functions, especially in response to intracellular ROS, the mutations in *trn*V — along with sequence changes in *HSP1* and *HSP2* — likely reflect adaptations to different intracellular environments and mitochondrial responses across strains. The presence of these incomplete tRNA fragments and the conservation of tRNA reveal the evolution of a specific region around the *atp6-1* and its copy *atp6-2*. Despite the addition of *HSP2* at the 5’ end of *atp6-2*, multiple tRNAs are still maintained in the same type and order as *atp6-1*.

### ﻿Phylogeny and evolution

**Figure 3.**
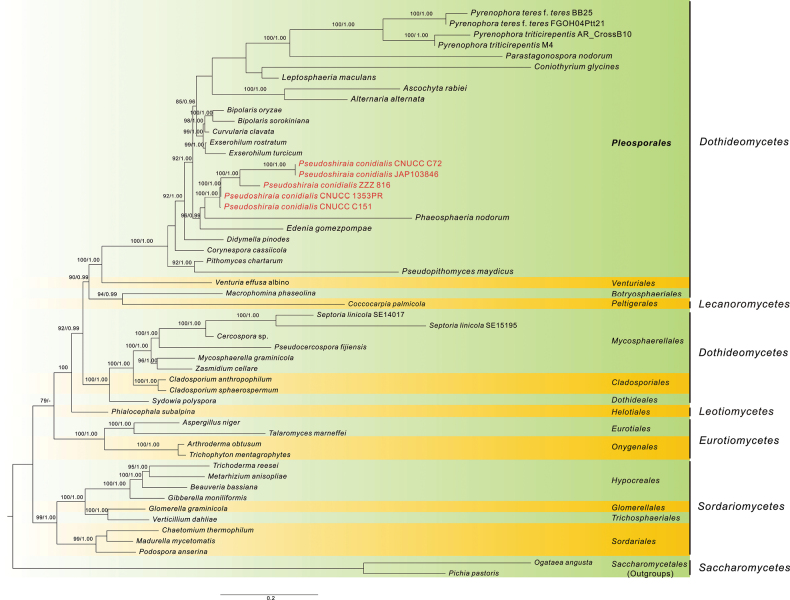
Phylogenetic tree derived from maximum likelihood analysis of combined 14 protein-coding genes (PCGs) of *Dothideomycetes* and the neighbor class, using *Ogataeaangusta* and *Pichiapastoris* as outgroups. Bootstrap support values for ML analysis (MLB) greater than 75% and Bayesian posterior probabilities (PP) greater than 0.95 are given above the nodes. Names of the *Shiraia*-like species have been written in red.

The phylogenetic status of *Shiraia* species were analysed by the combined PCGs from mitochondria and Bayesian Inference (BI) and Maximum Likelihood (ML) were employed to construct the phylogenetic tree of *Dothideomycetes* and the neighbour classes (*Eurotiomycetes*, *Lecanoromycetes*, *Leotiomycetes* and *Sordariomycetes*) (Fig. 3). Most of the major clades were well supported (BPP ≥ 0.99; BS = 100) and the species of *Shiraia*-like showed a disparate phylogenetic relationship with the *Pleosporales* species and consisted of a unique and diverse group. It is noteworthy of some close species to display uneven relationships between them by different single PCGs and more sophisticated in consideration of the plant-pathogenic interaction and hypocrellin synthesisation (Lu et al. 2024).

The phylogenetic tree revealed that CNUCC C151, which contained *HSP1*, but lacked *HSP2* and *atp6_2*, occupies the basal position amongst *Shiraia*-like species. This strain is adjacent to CNUCC 1353PR and ZZZ816, possessing the complete *HSP1-HSP2-atp6_2* segment and the group of JAP103846 and CNUCC C72, which have lost this unit (Fig. 2). This taxonomic arrangement suggests that CNUCC C151 with its *HSP1* gene emerged initially, followed by ZZZ816 and CNUCC 1353PR strains containing duplicated *atp6_2* and *HSP2*, while JAP103846 and CNUCC C72 subsequently lost the entire *HSP1*-*HSP2*-*atp6_2* segment.

### ﻿Gain/loss of a copied *atp6* gene, two *HSPs* and three trns

The *HSP1_HSP2_atp6-2* fragment appears between *nad3* and *trn*V from ZZZ816 and CNUCC1353PR and undergoes significant changes in comparative mitogenomes, while the surrounding coding genes with most intergenic regions remain stable. There are a copied *atp6* gene (*atp6_2*), two *HSPs* (*HSP1* and *HSP2*) and three *trns* (*trn*Q, *trn*H and *trn*M) in this section (Fig. 2). By the comparative mitogenomes, strains JAP103846 and CNUCC C72 present the minimal gene content and do not contain any genes between *nad3* and *trn*V, where CNUCC C151 had the acquisition of *HSP1* at this location and only strains CNUCC1353PR and ZZZ816 had the complete segment with duplication of *atp6*, both of *HSP1* and *HSP2* and a re-location of *trns* Q-H-M. The *atp6-2* gene in this section belongs to the copy of the right *atp6* and, compared to *atp6-1*, *atp6-2*, shows partial sequence deletions at the 5’ end and the addition of extra sequence at the 3’ end (Suppl. material 3). Through detailed sequence alignment, the missing 5’ end portion seems to be located on a remote location, where the existence of *HSP2* has hindered the direct observation (Suppl. material 3). Furthermore, our transcription analysis shows that *atp6_2* possesses its own AUG start codon and the similar 5’ end region cannot be translated into an amino acid sequence with effective length.

*HSP1* is rarely found in the relevant species from the same order *Pleosporales* and class *Dothideomycetes* and the classification of *HSP2* also conflicts with the phylogenetic tree mentioned above. The elaborate analysis of the *HSP1_ HSP2_atp6-2* segment allows the species around *Shiraia*-like to be divided into three groups. ZZZ816 and CNUCC1353PR retain the complete part of the *HSP1_atp6-2* region, where *HSP1*, *HSP2* and *atp6-2* are all located. JAP10346 and CNUCC C72 have completely lost this fragment and the three related genes cannot be found in the mitogenome. CNUCC C151 retains a partial region, covering the complete sequence of *HSP1* gene.

Analysis of the conserved region of HSP1 revealed that the C-terminal sequence was similar to Mer2 and YtxH-like, which might be involved in the formation of intracellular cytoskeleton and membrane. After mining the public mitogenome database, the results suggested that the protein tended to be regarded as the partial region of NAD5. Homologous genes of *Bipolarisoryzae* and *Edeniagomezpompae*, from *Pleosporales*, showed higher identity with HSP1, but unexpectedly, NAD5 from the original mitogenome of *Shiraia* had a more distant relationship (Deng et al. 2019; Huang et al. 2022).

The closest annotated homologue of HSP2 belongs to the sec-independent transporter family. This homologue has been identified in *Saprolegniaparasitica* (Jiang et al. 2013) and *Saprolegniaferax* (Grayburn et al. 2004), with only 26% similarity and 51.1% coverage. The sec-independent transporter protein functions without the sec protein and has homologues in bacterial, mitochondrial and chloroplast plasma membranes, though its precise role in fungi remains unclear. Additionally, whether in the same or parallel class, there were almost no homologous genes with over moderate or high similarity in existing mitogenomes, except for a sequence-similar protein in *Exserohilumturcicum*. It is noteworthy that the mitogenome of *Exserohilumrostratum* (*E.turcicum*-like species) did not contain the related protein region (Ma et al. 2022). The hypothetical protein J6642_mgp09 from *Pisolithusmicrocarpus* of *Agaricomycetes* has been singled out with the highest identity (Wu et al. 2021). The similar protein in *Tetranychusurticae* of Metazoa was even misidentified as the portion of *atp6* (van Leeuwen et al. 2008). Correspondingly, the homologous protein was usually explored around the *atp6* gene, such as the hypothetical protein M1I11_mgp149 from *E.turcicum* (Ma et al. 2022).

Furthermore, it is interesting that in the “*HSP1*-*HSP2*-*atp6_2*” segment, the order and sequences of *trn*Q, *trn*H and *trn*M between *HSP1* and *HSP2* show high similarity with the neighbouring region at the 5’ end of *atp6_1* (Suppl. material 3). This suggests that these *trns* likely underwent relocation together with the *atp6* gene. The positioning of *HSP2* between these three tRNAs and *atp6_2* indicates that *HSP2*’s insertion might occur after the duplication of *atp6*.

In this study, we have screened nearly all strain types currently employed in the fermented production. Amongst them, JAP10346 types require the supplement of inducers, such as Tween 80 to activate the biosynthetic progress of hypocrellin and enhance the final yield of these natural products (Vu et al. 2010). On the other hand, ZZZ816 types exhibit a high yield at the original stage and can be directly applied in fermented production without further modification (Shen et al. 2014; Liu et al. 2016; Lu et al. 2024). The mini-evolution characteristic of the *HSP1_HSP2_atp6-2* fragment indicates a close relationship amongst the original strains that have different production capacities. Strains JAP10346 and CNUCC C72, which lack the *HSP1_HSP2_atp6-2* fragment and its associated coding genes, are generally unable to produce hypocrellin effectively on their own without media optimisation or strain modification. In contrast, ZZZ816 and CNUCC1353PR strains have sufficient yield of hypocrellin at the initial stage, which can be observed directly from the phenotypic red pigment. CNUCC C151, located in the middle state, also displays a certain synthesis capacity under temperate conditions.

### ﻿Evolutionary characteristics of gene adaptation

In general, *atp6* is considered as a key functional gene in mitochondria, playing a crucial role in complex V of the respiratory chain. It is involved in ATP synthesis, mitochondrial DNA replication and maintenance of mitochondrial structural stability (Kabala et al. 2022). Subtle differences in the conserved region of *atp6* can be used as molecular markers for taxonomic identification and phylogenetic analysis, as well as determining genetic relationships and population structure within species (Ben Slimen et al. 2017; Zhang et al. 2023; Li et al. 2024). The analysis of *atp6* gene sequences has been widely employed in animal taxonomy and participated in the classification of fungi (Ben Slimen et al. 2018; Nkurikiyimfura et al. 2024; Zhang et al. 2024). In the plant kingdom, *atp6* genes sometimes appear as multiple copies and play a role in genetic breeding and crop improvement (Zancani et al. 2020; Gowd et al. 2022). In *Arabidopsisthaliana*, the copies of *atp6* genes are functionally redundant and can complement each other (Arimura et al. 2020). In fungi, homologous *atp6* genes seldom arise in few species and have not received much attention (Kabala et al. 2022; Nkurikiyimfura et al. 2024). In the mitogenomes of *Shiraia*-like species, the copy *atp6* gene *atp6-2* is found in ZZZ816 and CNUCC1353PR, along with two unknown functional genes, *HSP1* and *HSP2*. A relevant case is observed in the neighbouring species *E.turcicum* and *E.rostratum* (Ma et al. 2022). Although the two mitogenomes from them have different sequence lengths, the comparative analysis still reveals there are highly-conserved gene arrangements and high identity sequence alignments and only a single *atp6* gene appears in each species (Ma et al. 2022). It is interesting that the location of *atp6* in *E.turcicum* is similar to *atp6-2* from ZZZ816, while the one in *E.rostratum* is indicated as *atp6-1*. Furthermore, the hypothetical protein named M1I11_mgp149, identified as a homologous protein of HSP2, only appears in *E.turcicum* and is settled next to the 5’ end of *atp6* (similar to *atp6-2*) with also a very similar gene sorting. This has not been explored in *E.rostratum* and the other close species from *Dothideomycetes* (Ma et al. 2022).

### ﻿Mitochondrial transcription of PCGs

PCGs often focus on analysing the genomic structure, but neglect the transcriptomic analysis, especially from the fungal kingdom. However, for *Shiraia*-like species, it is important to study the expression of these mitochondrial genes under different conditions. By the comparative mitogenomes above, it was found that the changes in genomic DNA were associated closely with strain characteristics and hypocrellin production, prominently of the combined fragment of three genes *HSP1*_*HSP2*_*atp6-2* (as shown from Suppl. material 2). Based on this, a comprehensive consideration of genomic structure and hypocrellin synthesis, could directly classify the available transcriptomes into two main types: ZZZ816 and a closely-related species and the JAP103846 associated group. Therefore, the two representative mitogenomes (ZZZ816 and JAP103846) were chosen as references for further transcriptional analysis.

These transcriptome data were collected from various treatment conditions, including a comparison between basic media (Czapek’s medium) and complete media (PDA), the addition of surfactant TritonX100, the use of the methylation reagent 5_azacytidine, exposure to blue light irradiation, changes in L-Arg content in the medium composition and induction of oxidative stress by SNP (Lei et al. 2017; Ma et al. 2018; Ma et al. 2021; Chen et al. 2022; Li et al. 2022). By the calculation of mapping efficiency, most of these samples were located on the species around JAP103846 group. Therefore, to compensate for the scarcity of ZZZ816 data, a time series expression analyses was conducted at different fermentation stages.

### ﻿Effects of different culture media components on hypocrellin synthesis and PCGs expression

Within the optimisation of media components, PDA media are used to improve hypocrellin production significantly, while Cz media display a negative effect. In this study, we have employed these two media to examine the expression of functional genes in the mitogenomes of ZZZ816 and JAP103846 separately and the samples derived from Cz media were designated as the control group (Suppl. material 4: fig. S4A). When the nutrient content became sufficient (PDA), both *atp6* genes in strain ZZZ816 were significantly down-regulated, with *atp6-2* showing greater inhibition and the expression of *HSP1* and *HSP2* was also significantly inhibited under PDA (Suppl. material 4: fig. S4A). On the other hand, the expression of *cox1-3* genes was significantly up-regulated, where *cox1* exhibited the most significant activation. However, the changes in expression of the *nad* family varied diversely: *nad1*, *nad3* and *nad6* showed significant down-regulation, while *nad4L* and *nad5* indicated significant up-regulation. Additionally, *rps3* was significantly inhibited, making it to be the most down-regulated one. The expression changes of PCGs from JAP103846 were also analysed in the same way (Suppl. material 4: fig. S4A). When the nutrient content was sufficient, the expression of *ATP6-1* and *cob* genes in JAP103846 was significantly inhibited, while the expression of *cox1-3* genes was significantly up-regulated. The expression of *nad1* and *nad3* was down-regulated to a greater extent. Although the overall expression of 12 functional genes was less affected by culture component variation, the total expression trend from JAP103846 was generally consistent with ZZZ816.

### ﻿Ethanol stress on hypocrellin synthesis and mitochondrial functional genes

Under ethanol stress, the changes in hypocrellin production were measured in strains ZZZ816 and JAP103846 in both basic and enriched culture media. It was found that both high-yield and low-yield strains showed significant inhibition in hypocrellin synthesis after treatment with 1% ethanol. Remarkably, the high-yield strain ZZZ816 showed a more sensitive response to ethanol. Through nutrient-rich PDA culture, a comparison of strain ZZZ816 before and after treatment showed that *atp6-1* and *nad2* were significantly up-regulated, while the expression of *cox1/2*, *HSP3* and *nad4/5* was significantly inhibited (Suppl. material 4: fig. S4A). Three genes, *atp6-2*, *HSP1* and *HSP2*, did not show significant expression changes under these conditions. In the basic culture (Cz media), the same strain ZZZ816 exhibited a different expression pattern (Suppl. material 4: fig. S4A). The *atp6-2* gene showed a definite down-regulation, while *cox1*, *nad2* and *nad4L* displayed a significant up-regulation. Interestingly, *atp6-2*, *cox1* and *nad4L* exhibited similar expression patterns to those observed in the previous variation of medium components. Therefore, it is suggested that the addition of ethanol under nutrient-deficient conditions may not only represent a stress treatment, but also serve as a carbon source supplementation. The expression patterns of PCGs in strain JAP103846 were analysed in the same way (Suppl. material 4: fig. S4A). In PDA media, only *nad6* showed a significant down-regulation, while *atp6-1* did not show significant change. Conversely, *cox1*, *nad2* and *nad4L* had the significant up-regulation, which differed from strain ZZZ816. In Cz media, the expression of *cox2* and *rps3* was significantly inhibited, *atp6-1* displayed an appropriate increase in expression and *nad2* and *nad4L* indicated a definite up-regulation. Above all, under ethanol stress conditions, strain ZZZ816 and JAP103846 exhibited different expression patterns of PCGs. However, *nad2* consistently showed a high expression and its sensitive response would contribute to the understanding on PCGs around stress resistance mechanisms.

The differential gene expression observed under hypocrellin and ethanol stress may be a result of complex regulatory networks that respond to cellular stress by modulating the expression of specific genes. This modulation could be a strategic response to maintain cellular energy homeostasis under stress conditions, with certain genes being up-regulated to compensate for the down-regulation of others.

We have recognised the need for a more comprehensive mechanistic understanding of these observations and our future research will focus on investigating the roles of transcription factors, post-transcriptional modifications and potential oxidative stress-induced protein interactions that may influence the expression levels of these genes.

### ﻿*Analysis of the expression of mitochondrial functional genes under different environmental factors*

In this study, we collected and screened almost all available transcriptomes related to *Shiraia*. Notably, the majority of these data belonged to the JAP103846 group and also had a close relationship with the synthesis of hypocrellin. Furthermore, these data could be categorised, based on different treatment conditions: 5-azacytidine (5-AC), Blue light (BL), L-arginine (Arg), sodium nitroferricyanide (SNP) and Triton X100 (Suppl. material 4: fig. S4B).

5-AC is generally regarded as a DNA methyltransferase inhibitor, playing an important role in methylation modification of fungal genome. For *Shiraia*-like species, the presence of this reagent can significantly inhibit the synthesis of hypocrellin and contribute to the significant activation of *atp6-1*, as well as the *nad* family genes *nad1*, *nad4L* and *nad5* (Suppl. material 4: fig. S4B).

Visible light, especially BL, can promote hypocrellin synthesis in *Shiraia*-like species. The PCGs in the mitogenome were globally detected (Suppl. material 4: fig. S4B) and multiple genes from the *nad* family displayed different responses. Specifically, *nad1* and *nad4L* showed significant increases in expression, while *nad4* expression was significantly inhibited. Noteworthy, the expression of *rps3* was continuously down-regulated during BL-induced hypocrellin production.

L-arginine (Arg), an amino acid nutrient component for the culture media, can enhance efficiency of hypocrellins synthesis by activating the NO signalling pathway. When examining the expression of PCGs (Suppl. material 4: fig. S4B), we observed pronounced up-regulation of *cox1* and down-regulation of *nad4*, while the transcriptional level of other PCGs remained relatively stable.

SNP, belonging to the nitroprusside type of compounds, acts as a donor of NO. It can assist in increasing hypocrellin production also by triggering the NO-cGMP-PKG signalling pathway. Unlike others, under SNP treatment, there was a significant increase in hypocrellin production, but no significant changes were observed from the expression of mitochondrial PCGs (Suppl. material 4: fig. S4B).

Triton X100, a surfactant, plays a crucial role in improving hypocrellin yield. With the activation of hypocrellin synthesis, the expression of *cox1-3* was significantly inhibited, while *nad3* and *nad4L* from the *nad* gene family were sincerely decreased and increased, respectively. The transcripts of *nad5* also showed a relative increase (Suppl. material 4: fig. S4B).

Through a comprehensive analysis of these factors, it was observed that they all had a close relationship with hypocrellin synthesis. However, the response of mitochondrial PCGs showed diverse patterns. This is likely due to the complicated metabolic pathways and signal transduction modes within the mycelial cells. The NO pathway was found to have less involvement with the PCGs of mitochondria, as most genes did not show sincere expression changes under L-Arg and SNP. In other words, the genes of the *nad* family are more sensitive to these stresses in the JAP103846 strain. Particularly, the expression of *nad4L* undergoes noticeable changes under multiple factor treatments. It is interesting to explore that the expression of *nad4L* increases along with enhanced hypocrellin production. However, unusually, when hypocrellin synthesis is inhibited by 5-AC, the associated expression also significantly increases, as well as *nad1*. Therefore, it is suggested that 5-AC participates in the epigenetic modification of cells through methylation, which is different from conventional stresses. Two separate responsive ways may activate the identical gene through different regulatory mechanisms.

### ﻿Synthesis of hypocrellin during different fermentation stages and expression changes of mitochondrial genes

By conducting a comprehensive analysis of existing data, it was observed that the JAP103846 group accounted for the majority, while the ZZZ816 group appeared infrequently. As a result, we specifically screened hypocrellin synthesis at different fermentation stages from ZZZ816 and combined them with the expression of PCGs (Suppl. material 4: fig. S4C; Suppl. material 5). The yield of hypocrellin at different stages was calculated from the beginning to the end of fermentation (36 h–264 h), covering from the lag phase to death phase, with seven representative stages: 36 h, 48 h, 60 h, 72 h, 108 h, 132 h and 264 h.

As shown in Suppl. material 5, hypocrellin production increased during the early stage of fermentation, reaching its peak at 108 hours and then gradually decreased until the end. Correspondingly, transcriptome analysis was employed to investigate the expression of PCGs during the same stages, with the initial stage (36 h) serving as the control group (Suppl. material 4: fig. S4C).

During the process, *atp6-1* and *atp6-2* showed completely opposite trends. In the early stage (48 h), both of them did not show significant changes. However, as the fermentation time extended (60 h), the expression of *atp6-1* remained suppressed, while *atp6-2* increased and maintained a high level from 60 h onwards, continuing on to the end (264 h). This unique divergent expression pattern has not been observed from other conditions.

The expression of *HSP1* and *HSP2* was significantly activated during hypocrellin synthesis. Especially *HSP2*, which maintained a high level of expression since 60 h of fermentation, indicated a change pattern consistent with that of *atp6*. Additionally, *cox1* and *cox2* exhibited early high expression at the beginning of hypocrellin production (48 h), while the expression of *rps3* remained suppressed during the same time (48 h–264 h).

As the high hypocrellin-producing strain, ZZZ816 begins to synthesise hypocrellin after 48 h at the earliest and the content in these mycelia can accumulate to a certain concentration at 60 h (Suppl. material 5). Therefore, by considering the comprehensive expression of PCGs at different fermentation stages, it is suggested that *cox1*, *cox2* and *rps3* may be closely related to hypocrellin synthesis, while the genes *atp6*, *HSP1* and *HSP2* are likely to be involved in enhancing tolerance to adverse stress (Suppl. material 4: fig. S4C), particularly resisting ROS caused by high concentrations of hypocrellin inside mycelial cells (Al Subeh et al. 2022; Liu et al. 2022).

In addition, some genes such as *nad2* and *nad4L*, showed significant expression changes during hypocrellin synthesis, but their activation or inhibition patterns at different time points were more flexible and diverse, indicating their potential involvement in a more complex regulatory mechanism. More treatment methods and time points are expected to be applied to unravel these regulatory pathways.

In summary of the transcriptional analysis, the ZZZ816 and JAP103846 strains display different expression changes in mitochondrial PCGs. It is notable that *ATP6*, *HSP1* and *HSP2* have potential roles in hypocrellin synthesis and the resistance of *Shiraia*-like species to various stresses. Additionally, *rps3* is known to play a crucial role in protein synthesis, cell growth and proliferation. It is also involved in cell apoptosis and DNA repair, which impact cell survival and stability (Graifer et al. 2014; Graifer and Karpova 2020). In the ZZZ816 strain, the expression of *rps3* is closely related to hypocrellin synthesis. Typically, as hypocrellin synthesis increases, the expression of *rps3* is always suppressed at a lower level. However, the JAP103846 strain does not exhibit the similar pattern, except for BL significantly inhibiting the expression of *rps3*. Other factors that promote an increase in hypocrellin production from JAP103846 do not have a significant effect on the expression of *rps3*. It is speculated that, in the high-yield strain ZZZ816, the global regulation of metabolic pathways has been altered and the expression of *rps3* contributes to improving tolerance to ROS from hypocrellin. In contrast, the low-yield strain JAP103846 is only forced to activate the higher-level regulation by the irradiation of BL.

Several nuclear genes within the specific cluster are involved in the hypocrellin biosynthetic pathway. Polyketide synthases (PKS) are essential for assembling the perylenequinone backbone, while cytochrome P450 mono-oxygenases add functional groups to hypocrellin molecules (Zhao et al. 2020). The transcription factor *SbTF* significantly influences the expression of these downstream genes, thereby regulating hypocrellin production (Lu et al. 2024). In future studies, we aim to combine mitochondrial and nuclear genes to better understand the genetic basis of hypocrellin biosynthesis in *Pseudoshiraiaconidialis*.

## ﻿Additional information

### Author ORCIDs

Xiao-Ye Shen https://orcid.org/0000-0003-0789-6850

Xue-Ting Cao https://orcid.org/0009-0007-9036-632X

Lan Zhuo https://orcid.org/0000-0002-8129-2812

Hui-Meng Yang https://orcid.org/0009-0002-5118-5618

Li Fan https://orcid.org/0000-0001-9887-7086

Cheng-Lin Hou https://orcid.org/0000-0001-8162-5560

### Data availability

The newly-isolated strains proposed in this study were preserved at the China Forestry Culture Collection Center (CFCC) and Capital Normal University Culture Collection Center (CNUCC), with the accession numbers provided in National Genomics Data Center (NGDC).

## Supplementary materials

Supplementary material 1 The comparison of morphological characteristics amongst distinct strains in PDA at 6^th^ day Data type jpg https://binary.pensoft.net/file/1256502This dataset is made available under the Open Database License (http://opendatacommons.org/licenses/odbl/1.0/). The Open Database License (ODbL) is a license agreement intended to allow users to freely share, modify, and use this Dataset while maintaining this same freedom for others, provided that the original source and author(s) are credited.Xiao-Ye Shen, Xue-Ting Cao, Xiao-Bo Huang, Lan Zhuo, Hui-Meng Yang, Li Fan, Cheng-Lin Hou

Supplementary material 2 The diversity of fragments between position *nad4L* and position *nad3* from the mitogenomes of *Shiraia*-like species Data type jpg https://binary.pensoft.net/file/1256503This dataset is made available under the Open Database License (http://opendatacommons.org/licenses/odbl/1.0/). The Open Database License (ODbL) is a license agreement intended to allow users to freely share, modify, and use this Dataset while maintaining this same freedom for others, provided that the original source and author(s) are credited.Xiao-Ye Shen, Xue-Ting Cao, Xiao-Bo Huang, Lan Zhuo, Hui-Meng Yang, Li Fan, Cheng-Lin Hou

Supplementary material 3 The direct comparison of the gene structures of *atp6-1* and *atp6-2* from ZZZ816 Data type jpg https://binary.pensoft.net/file/1256504This dataset is made available under the Open Database License (http://opendatacommons.org/licenses/odbl/1.0/). The Open Database License (ODbL) is a license agreement intended to allow users to freely share, modify, and use this Dataset while maintaining this same freedom for others, provided that the original source and author(s) are credited.Xiao-Ye Shen, Xue-Ting Cao, Xiao-Bo Huang, Lan Zhuo, Hui-Meng Yang, Li Fan, Cheng-Lin Hou

Supplementary material 4 The differential expression of mitochondrial genes Data type jpg Explanation note (a) The differential expression of mitochondrial genes from ZZZ816 and JAP103846 under distinct media conditions and ethanol stress, including Czapek-Dox (Cz) and Potato Dextrose Agar (PDA), Ethanol stress on PDA media and Ethanol stress on Cz media; (b) The differential expression of mitochondrial genes from JAP103846 on different treatment conditions: 5-azacytidine (5-AC), Blue light (BL), L-arginine (Arg), sodium nitroferricyanide (SNP) and Triton X100; (c) The transcriptional expression of PCGs during seven representative stages (36 h, 48 h, 60 h, 72 h, 108 h, 132 h and 264 h), with the initial stage (36 h) serving as the control group. https://binary.pensoft.net/file/1256505This dataset is made available under the Open Database License (http://opendatacommons.org/licenses/odbl/1.0/). The Open Database License (ODbL) is a license agreement intended to allow users to freely share, modify, and use this Dataset while maintaining this same freedom for others, provided that the original source and author(s) are credited.Xiao-Ye Shen, Xue-Ting Cao, Xiao-Bo Huang, Lan Zhuo, Hui-Meng Yang, Li Fan, Cheng-Lin Hou

Supplementary material 5 The hypocrellin per unit yield of typical stages Data type jpg Explanation note The hypocrellin per unit yield of typical stages was calculated from the beginning to the end of fermentation (36 h–264 h), covering from the lag phase to death phase: 36 h, 48 h, 60 h, 72 h, 84 h, 96 h, 108 h, 120 h, 132 h, 144 h and 264 h. https://binary.pensoft.net/file/1256506This dataset is made available under the Open Database License (http://opendatacommons.org/licenses/odbl/1.0/). The Open Database License (ODbL) is a license agreement intended to allow users to freely share, modify, and use this Dataset while maintaining this same freedom for others, provided that the original source and author(s) are credited.Xiao-Ye Shen, Xue-Ting Cao, Xiao-Bo Huang, Lan Zhuo, Hui-Meng Yang, Li Fan, Cheng-Lin Hou

Supplementary material 6 Supplementary tables Data type docx Explanation note **table S1**: Organisation and features of JAP103846 mitochondrial genome; **table S2**: Organisation and features of CNUCC C72 mitochondrial genome; **table S3**: Organisation and features of ZZZ816 mitochondrial genome; **table S4**: Organisation and features of CNUCC1353PR mitochondrial genome; **table S5**: Organisation and features of CNUCC C151 mitochondrial genome. https://binary.pensoft.net/file/1256507This dataset is made available under the Open Database License (http://opendatacommons.org/licenses/odbl/1.0/). The Open Database License (ODbL) is a license agreement intended to allow users to freely share, modify, and use this Dataset while maintaining this same freedom for others, provided that the original source and author(s) are credited.Xiao-Ye Shen, Xue-Ting Cao, Xiao-Bo Huang, Lan Zhuo, Hui-Meng Yang, Li Fan, Cheng-Lin Hou
