# Supplementary figures and images for: ﻿Mitochondrial genome and transcription of Shiraia-like species reveal evolutionary aspects in protein-coding genes
Source: IMA Fungus. 2025 Feb 20;16:e138572. doi: 10.3897/imafungus.16.138572 (PMC11881002; doi:10.3897/imafungus.16.138572)

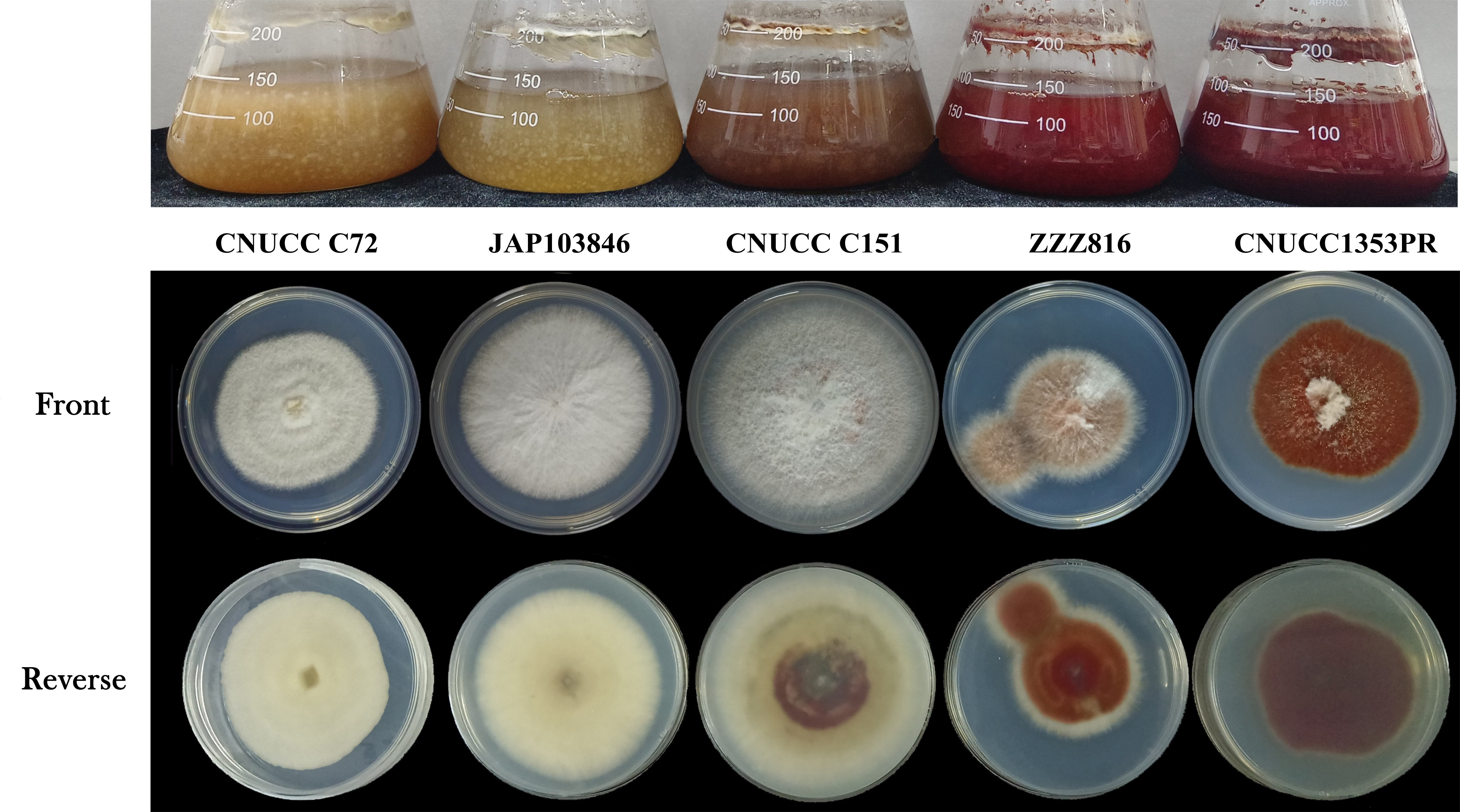

Supplement: Supplementary material 1 — The comparison of morphological characteristics amongst distinct strains in PDA at 6th day [file imafungus-16-e138572-s001.jpg]

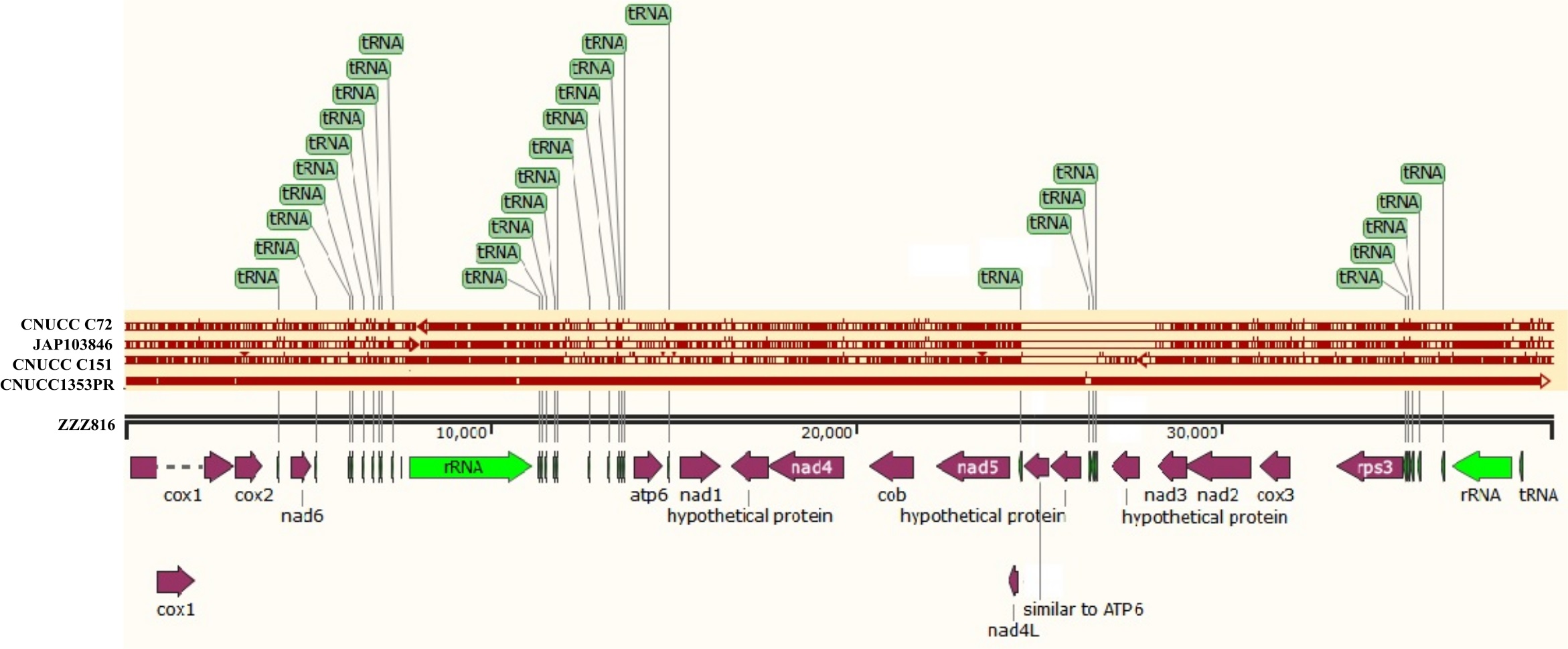

Supplement: Supplementary material 2 — The diversity of fragments between position nad4L and position nad3 from the mitogenomes of Shiraia-like species [file imafungus-16-e138572-s002.jpg]

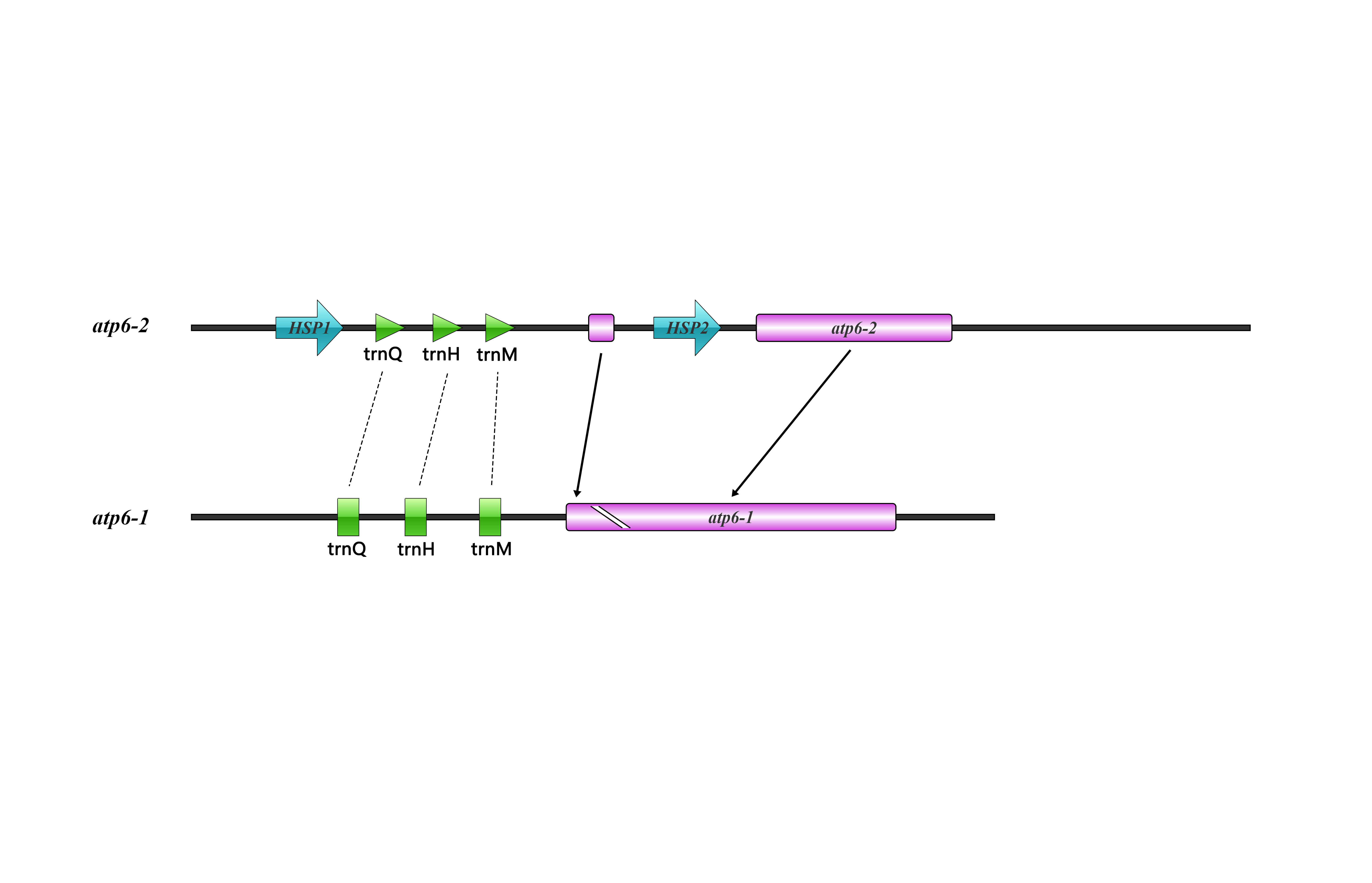

Supplement: Supplementary material 3 — The direct comparison of the gene structures of atp6-1 and atp6-2 from ZZZ816 [file imafungus-16-e138572-s003.jpg]

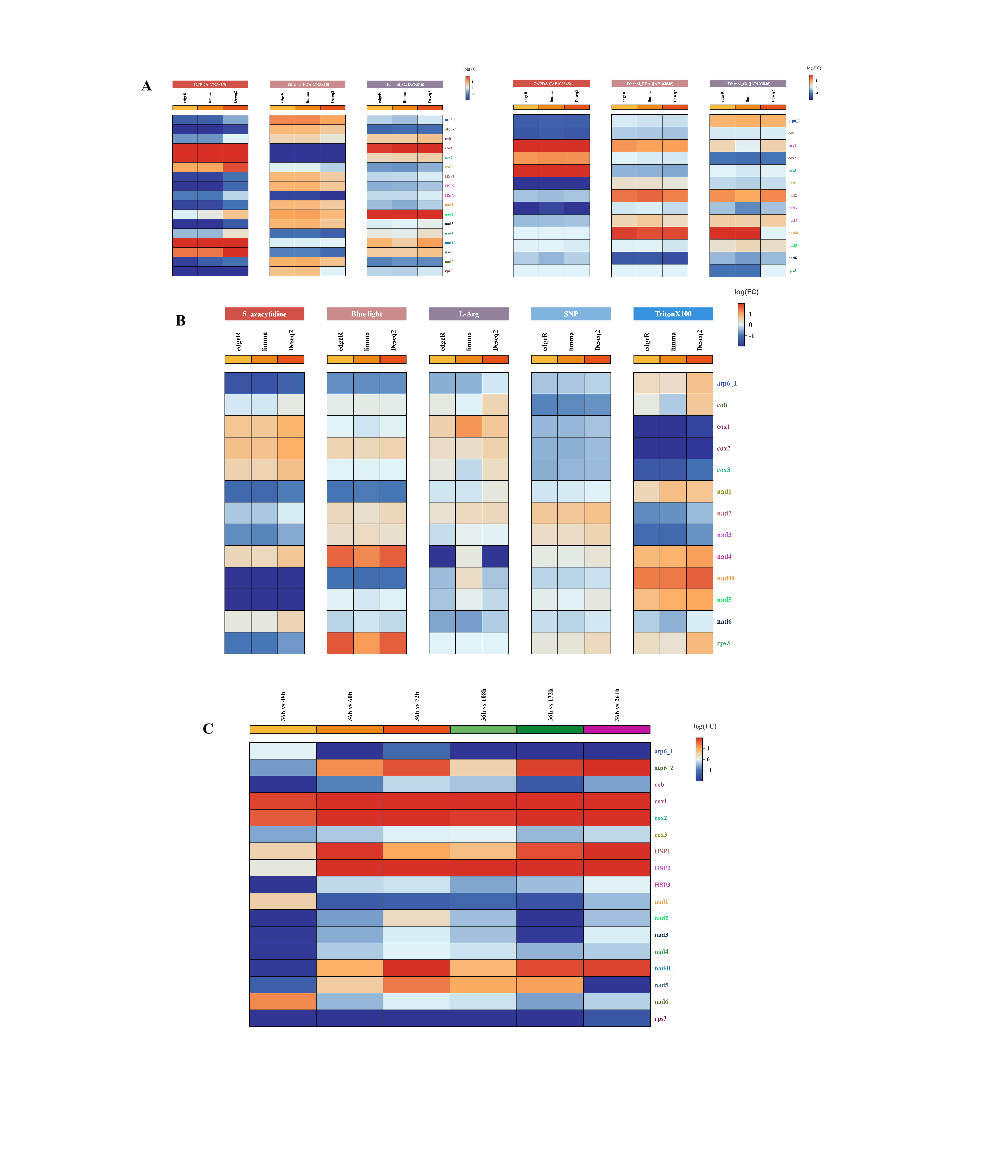

Supplement: Supplementary material 4 — The differential expression of mitochondrial genes [file imafungus-16-e138572-s004.jpg]

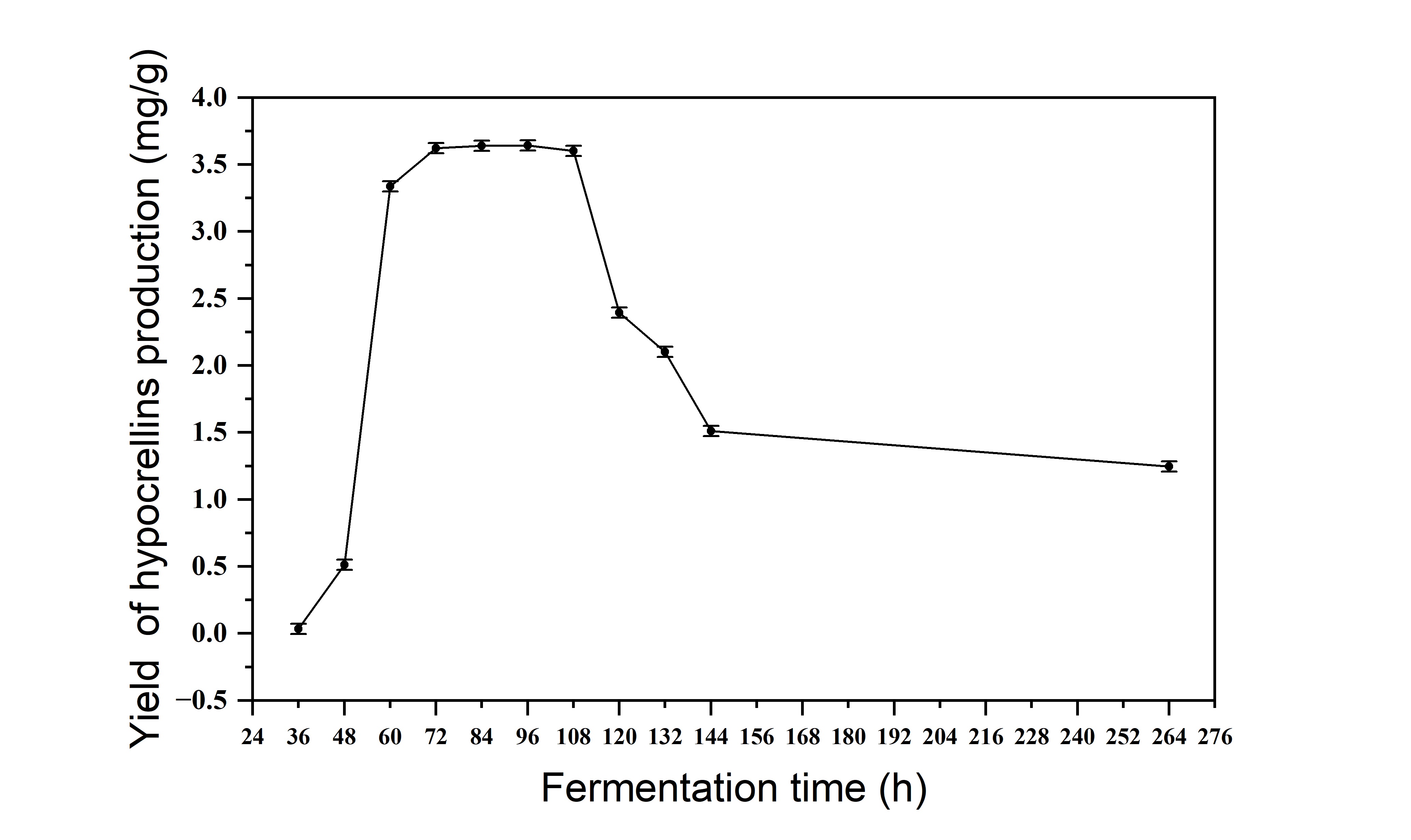

Supplement: Supplementary material 5 — The hypocrellin per unit yield of typical stages [file imafungus-16-e138572-s005.jpg]
