# Supplementary material for: ﻿Mitochondrial genome and transcription of Shiraia-like species reveal evolutionary aspects in protein-coding genes
Source: IMA Fungus. 2025 Feb 20;16:e138572. doi: 10.3897/imafungus.16.138572 (PMC11881002; doi:10.3897/imafungus.16.138572)
Supplement: Supplementary material 6 — Supplementary tables [file imafungus-16-e138572-s006.docx]

Supplementary Table 1. Organisation and features of JAP103846 mitochondrial genome.

| Gene | Start | Stop | Length (nt) | Length (aa) | Start Codon | Stop codon | Intron Start | Intron Stop | Intron type |
| --- | --- | --- | --- | --- | --- | --- | --- | --- | --- |
| *trn*T | 3222 | 3292 | 71 |  |  |  |  |  |  |
| *trn*M | 3315 | 3385 | 71 |  |  |  |  |  |  |
| *trn*M | 3391 | 3463 | 73 |  |  |  |  |  |  |
| *trn*E | 3640 | 3712 | 73 |  |  |  |  |  |  |
| *trn*A | 3745 | 3816 | 72 |  |  |  |  |  |  |
| *trn*F | 4579 | 4651 | 73 |  |  |  |  |  |  |
| *trn*L | 5043 | 5125 | 83 |  |  |  |  |  |  |
| *trn*Q | 5330 | 5401 | 72 |  |  |  |  |  |  |
| *trn*H | 5405 | 5478 | 74 |  |  |  |  |  |  |
| *trn*M | 5527 | 5598 | 72 |  |  |  |  |  |  |
| *atp6* | 5731 | 6500 | 770 | 256 | TTA | TAA |  |  |  |
| *trn*C | 6594 | 6665 | 72 |  |  |  |  |  |  |
| *nad1* | 6954 | 8069 | 1116 | 372 | ATG | TAA |  |  |  |
| Orf1 | 8323 | 9297 | 975 | 325 | TTA | CAT |  |  |  |
| *nad4* | 9339 | 11384 | 2046 | 682 | ATG | TAG |  |  |  |
| *cob* | 12128 | 13285 | 1158 | 386 | ATG | TAA |  |  |  |
| *nad5* | 13923 | 15908 | 1986 | 662 | ATG | TAA |  |  |  |
| *nad4L* | 15908 | 16177 | 270 | 90 | ATG | TAA |  |  |  |
| *trn*V | 16209 | 16281 | 73 |  |  |  |  |  |  |
| *nad3* | 16330 | 17097 | 768 | 256 | ATG | TAA |  |  |  |
| *nad2* | 17098 | 18885 | 1788 | 596 | ATG | TAA |  |  |  |
| *cox3* | 19137 | 19946 | 810 | 270 | ATG | TAA |  |  |  |
| *rps3* | 21176 | 22954 | 1779 | 593 | TTG | TAG |  |  |  |
| *trn*K | 22968 | 23039 | 72 |  |  |  |  |  |  |
| *trn*V | 23069 | 23141 | 73 |  |  |  |  |  |  |
| *trn*N | 23209 | 23279 | 71 |  |  |  |  |  |  |
| *trn*Y | 23375 | 23459 | 85 |  |  |  |  |  |  |
| *trn*K | 23983 | 24065 | 83 |  |  |  |  |  |  |
| *trn*R | 26050 | 26120 | 71 |  |  |  |  |  |  |
| *cox1* | 27770 | 28826 | 1057 | 352 | GTA | TAA |  |  |  |
| *cox2* | 29943 | 30686 | 744 | 248 | TTA | TAG |  |  |  |
| *trn*N | 31013 | 31083 | 71 |  |  |  |  |  |  |
| *nad6* | 31366 | 31953 | 588 | 196 | ATG | TAA |  |  |  |
| *trn*V | 32025 | 32097 | 73 |  |  |  |  |  |  |
| *trn*G | 32935 | 33007 | 73 |  |  |  |  |  |  |
| *trn*D | 33010 | 33081 | 72 |  |  |  |  |  |  |
| *trn*S | 33341 | 33420 | 80 |  |  |  |  |  |  |
| *trn*W | 33599 | 33670 | 72 |  |  |  |  |  |  |
| *trn*I | 33773 | 33844 | 72 |  |  |  |  |  |  |
| *trn*R | 33849 | 33920 | 72 |  |  |  |  |  |  |
| *trn*S | 34126 | 34210 | 85 |  |  |  |  |  |  |
| *trn*P | 34402 | 34474 | 73 |  |  |  |  |  |  |

Supplementary Table 2. Organisation and features of CNUCC C72 mitochondrial genome.

| Gene | Start | Stop | Length (nt) | Length (aa) | Start Codon | Stop codon | Intron Start | Intron Stop | Intron type |
| --- | --- | --- | --- | --- | --- | --- | --- | --- | --- |
| *trn*P | 333 | 405 | 73 |  |  |  |  |  |  |
| *trn*S | 597 | 681 | 85 |  |  |  |  |  |  |
| *trn*R | 890 | 961 | 72 |  |  |  |  |  |  |
| *trn*I | 966 | 1037 | 72 |  |  |  |  |  |  |
| *trn*W | 1140 | 1211 | 72 |  |  |  |  |  |  |
| *trn*S | 1390 | 1469 | 80 |  |  |  |  |  |  |
| *trn*D | 1729 | 1800 | 72 |  |  |  |  |  |  |
| *trn*G | 1803 | 1875 | 73 |  |  |  |  |  |  |
| *trn*V | 2713 | 2785 | 73 |  |  |  |  |  |  |
| *nad6* | 2857 | 3444 | 588 | 196 | ATG | TAA |  |  |  |
| *trn*N | 3727 | 3797 | 71 |  |  |  |  |  |  |
| *cox2* | 4124 | 4867 | 744 | 248 | TTA | TAG |  |  |  |
| *cox1* | 5984 | 7040 | 1057 | 352 | GTA | TAA |  |  |  |
| *trn*R | 8709 | 8779 | 71 |  |  |  |  |  |  |
| *trn*K | 10764 | 10846 | 83 |  |  |  |  |  |  |
| *trn*Y | 11370 | 11454 | 85 |  |  |  |  |  |  |
| *trn*N | 11550 | 11620 | 71 |  |  |  |  |  |  |
| *trn*V | 11689 | 11761 | 73 |  |  |  |  |  |  |
| *trn*K | 11791 | 11862 | 72 |  |  |  |  |  |  |
| *rps3* | 11876 | 13654 | 1779 | 593 | TTG | TAG |  |  |  |
| *cox3* | 14884 | 15693 | 810 | 270 | ATG | TAA |  |  |  |
| *nad2* | 15945 | 17732 | 1788 | 596 | ATG | TAA |  |  |  |
| *nad3* | 17733 | 18500 | 768 | 256 | ATG | TAA |  |  |  |
| *trn*V | 18549 | 18621 | 73 |  |  |  |  |  |  |
| *nad4L* | 18653 | 18922 | 270 | 90 | ATG | TAA |  |  |  |
| *nad5* | 18922 | 20907 | 1986 | 662 | ATG | TAA |  |  |  |
| *cob* | 21545 | 22702 | 1158 | 386 | ATG | TAA |  |  |  |
| *nad4* | 23446 | 25491 | 2046 | 682 | ATG | TAG |  |  |  |
| Orf_1 | 25533 | 26501 | 969 | 323 | ATG | TAA |  |  |  |
| *nad1* | 26749 | 27864 | 1116 | 372 | ATG | TAA |  |  |  |
| *trn*C | 28153 | 28224 | 72 |  |  |  |  |  |  |
| *atp6* | 28317 | 29086 | 770 | 256 | TTA | TAA |  |  |  |
| *trn*M | 29219 | 29290 | 72 |  |  |  |  |  |  |
| *trn*H | 29339 | 29412 | 74 |  |  |  |  |  |  |
| *trn*Q | 29416 | 29487 | 72 |  |  |  |  |  |  |
| *trn*L | 29692 | 29774 | 83 |  |  |  |  |  |  |
| *trn*F | 30210 | 30282 | 73 |  |  |  |  |  |  |
| *trn*A | 31045 | 31116 | 72 |  |  |  |  |  |  |
| *trn*E | 31149 | 31221 | 73 |  |  |  |  |  |  |
| *trn*M | 31398 | 31470 | 73 |  |  |  |  |  |  |
| *trn*M | 31476 | 31546 | 71 |  |  |  |  |  |  |
| *trn*T | 31569 | 31639 | 71 |  |  |  |  |  |  |

Supplementary Table 3. Organisation and features of ZZZ816 mitochondrial genome.

| Gene | Start | Stop | Length (nt) | Length (aa) | Start Codon | Stop codon | Intron Start | Intron Stop | Intron type |
| --- | --- | --- | --- | --- | --- | --- | --- | --- | --- |
| *cox1* | 162 | 3046 | 2885 | 540 | ATG | TAA | 893 | 2157 |  |
| *cox2* | 3047 | 3793 | 747 | 249 | ATG | TAG |  |  |  |
| *trn*N | 4169 | 4239 | 71 |  |  |  |  |  |  |
| *nad6* | 4542 | 5129 | 588 | 196 | ATG | TAA |  |  |  |
| *trn*V | 5201 | 5273 | 73 |  |  |  |  |  |  |
| *trn*G | 6116 | 6188 | 73 |  |  |  |  |  |  |
| *trn*D | 6191 | 6262 | 72 |  |  |  |  |  |  |
| *trn*S | 6502 | 6581 | 80 |  |  |  |  |  |  |
| *trn*W | 6761 | 6832 | 72 |  |  |  |  |  |  |
| *trn*I | 6934 | 7005 | 72 |  |  |  |  |  |  |
| *trn*R | 7010 | 7081 | 72 |  |  |  |  |  |  |
| *trn*S | 7288 | 7372 | 85 |  |  |  |  |  |  |
| *trn*P | 7564 | 7636 | 73 |  |  |  |  |  |  |
| *trn*T | 11299 | 11369 | 71 |  |  |  |  |  |  |
| *trn*M | 11392 | 11462 | 71 |  |  |  |  |  |  |
| *trn*M | 11468 | 11540 | 73 |  |  |  |  |  |  |
| *trn*E | 11719 | 11791 | 73 |  |  |  |  |  |  |
| *trn*A | 11824 | 11895 | 72 |  |  |  |  |  |  |
| *trn*F | 12675 | 12747 | 73 |  |  |  |  |  |  |
| *trn*L | 13201 | 13283 | 83 |  |  |  |  |  |  |
| *trn*Q | 13449 | 13530 | 72 |  |  |  |  |  |  |
| *trn*H | 13534 | 13607 | 74 |  |  |  |  |  |  |
| *trn*M | 13655 | 13726 | 72 |  |  |  |  |  |  |
| *atp6* | 13946 | 14719 | 774 | 258 | ATG | TAA |  |  |  |
| *trn*C | 14824 | 14895 | 72 |  |  |  |  |  |  |
| *nad1* | 15187 | 16302 | 1116 | 372 | ATG | TAA |  |  |  |
| *HSP3* | 16612 | 17580 | 969 | 323 | ATG | TAA |  |  |  |
| *nad4* | 17622 | 19667 | 2046 | 682 | ATG | TAG |  |  |  |
| *cob* | 20416 | 21573 | 1158 | 386 | ATG | TAA |  |  |  |
| *nad5* | 22205 | 24190 | 1986 | 662 | ATG | TAA |  |  |  |
| *nad4L* | 24190 | 24459 | 270 | 90 | ATG | TAA |  |  |  |
| *trn*V | 24491 | 24563 | 73 |  |  |  |  |  |  |
| *atp6-2* | 24599 | 25171 | 573 | 191 | ATG | TAA |  |  |  |
| *HSP2* | 25338 | 26126 | 789 | 263 | ATG | TAA |  |  |  |
| *trn*M | 26344 | 26415 | 72 |  |  |  |  |  |  |
| *trn*H | 26465 | 26538 | 74 |  |  |  |  |  |  |
| *trn*Q | 26642 | 26613 | 72 |  |  |  |  |  |  |
| *HSP1* | 26925 | 27677 | 753 | 251 | ATG | TAA |  |  |  |
| *nad3* | 28259 | 29026 | 768 | 256 | ATG | TAA |  |  |  |
| *nad2* | 29027 | 30814 | 1788 | 596 | ATG | TAA |  |  |  |
| *cox3* | 31074 | 31883 | 810 | 270 | ATG | TAA |  |  |  |
| *rps3* | 33188 | 34966 | 1779 | 593 | TTG | TAG |  |  |  |
| *trn*K | 34979 | 35050 | 72 |  |  |  |  |  |  |
| *trn*V | 35080 | 35152 | 73 |  |  |  |  |  |  |
| *trn*N | 35218 | 35288 | 71 |  |  |  |  |  |  |
| *trn*Y | 35384 | 35468 | 85 |  |  |  |  |  |  |
| *trn*K | 36038 | 36120 | 83 |  |  |  |  |  |  |
| *trn*R | 38172 | 38242 | 71 |  |  |  |  |  |  |

Supplementary Table 4. Organisation and features of CNUCC1353PR mitochondrial genome.

| Gene | Start | Stop | Length (nt) | Length (aa) | Start Codon | Stop codon | Intron Start | Intron Stop | Intron type |
| --- | --- | --- | --- | --- | --- | --- | --- | --- | --- |
| *cox1* | 118 | 2999 | 2882 | 539 | ATG | TTG | 849 | 2113 |  |
| *cox2* | 3006 | 3749 | 744 | 248 | TTA | TAG |  |  |  |
| *trn*N | 4125 | 4195 | 71 |  |  |  |  |  |  |
| *nad6* | 4498 | 5085 | 588 | 196 | ATG | TAA |  |  |  |
| *trn*V | 5157 | 5229 | 73 |  |  |  |  |  |  |
| *trn*G | 6072 | 6144 | 73 |  |  |  |  |  |  |
| *trn*D | 6147 | 6218 | 72 |  |  |  |  |  |  |
| *trn*S | 6458 | 6537 | 80 |  |  |  |  |  |  |
| *trn*W | 6717 | 6788 | 72 |  |  |  |  |  |  |
| *trn*I | 6890 | 6961 | 72 |  |  |  |  |  |  |
| *trn*R | 6966 | 7037 | 72 |  |  |  |  |  |  |
| *trn*S | 7244 | 7328 | 85 |  |  |  |  |  |  |
| *trn*P | 7520 | 7592 | 73 |  |  |  |  |  |  |
| *trn*T | 11255 | 11325 | 71 |  |  |  |  |  |  |
| *trn*M | 11348 | 11418 | 71 |  |  |  |  |  |  |
| *trn*M | 11424 | 11496 | 73 |  |  |  |  |  |  |
| *trn*E | 11675 | 11747 | 73 |  |  |  |  |  |  |
| *trn*A | 11780 | 11851 | 72 |  |  |  |  |  |  |
| *trn*F | 12631 | 12703 | 73 |  |  |  |  |  |  |
| *trn*L | 13157 | 13239 | 83 |  |  |  |  |  |  |
| *trn*Q | 13415 | 13486 | 72 |  |  |  |  |  |  |
| *trn*H | 13490 | 13563 | 74 |  |  |  |  |  |  |
| *trn*M | 13611 | 13682 | 72 |  |  |  |  |  |  |
| *atp6* | 13902 | 14675 | 774 | 258 | ATG | TAA |  |  |  |
| *trn*C | 14780 | 14851 | 72 |  |  |  |  |  |  |
| *nad1* | 15143 | 16258 | 1116 | 372 | ATG | TAA |  |  |  |
| Orf1 | 16568 | 17536 | 969 | 323 | TTA | CAT |  |  |  |
| *nad4* | 17578 | 19623 | 2046 | 682 | ATG | TAG |  |  |  |
| *trn*V* | 19959 | 19988 | 30 |  |  |  |  |  |  |
| *cob* | 20372 | 21529 | 1158 | 386 | ATG | TAA |  |  |  |
| *nad5* | 22161 | 24146 | 1986 | 662 | ATG | TAA |  |  |  |
| *nad4L* | 24146 | 24415 | 270 | 90 | ATG | TAA |  |  |  |
| *trn*V | 24447 | 24519 | 73 |  |  |  |  |  |  |
| *atp6-2* | 24645 | 25199 | 555 | 185 | GTA | CAT |  |  |  |
| Orf2 | 25294 | 26082 | 789 | 263 | TTA | CAT |  |  |  |
| *trn*M | 26302 | 26373 | 72 |  |  |  |  |  |  |
| *trn*H | 26421 | 26494 | 74 |  |  |  |  |  |  |
| *trn*Q | 26498 | 26569 | 72 |  |  |  |  |  |  |
| Orf3 | 26881 | 27633 | 753 | 251 | TTA | CAT |  |  |  |
| *nad3* | 28215 | 28982 | 768 | 256 | ATG | TAA |  |  |  |
| *nad2* | 28983 | 30770 | 1788 | 596 | ATG | TAA |  |  |  |
| *cox3* | 31030 | 31839 | 810 | 270 | ATG | TAA |  |  |  |
| *rps3* | 33144 | 34922 | 1779 | 593 | TTG | TAG |  |  |  |
| *trn*K | 34935 | 35006 | 72 |  |  |  |  |  |  |
| *trn*V | 35036 | 35108 | 73 |  |  |  |  |  |  |
| *trn*N | 35174 | 35244 | 71 |  |  |  |  |  |  |
| *trn*Y | 35340 | 35424 | 85 |  |  |  |  |  |  |
| *trn*K | 35994 | 36076 | 83 |  |  |  |  |  |  |
| *trn*R | 38128 | 38198 | 71 |  |  |  |  |  |  |

Supplementary Table 5. Organisation and features of CNUCC C151 mitochondrial genome.

| Gene | Start | Stop | Length (nt) | Length (aa) | Start Codon | Stop codon | Intron Start | Intron Stop | Intron type |
| --- | --- | --- | --- | --- | --- | --- | --- | --- | --- |
| Orf1 | 34 | 786 | 753 | 251 | ATG | TAA |  |  |  |
| *trn*V* | 1029 | 1100 | 72 |  |  |  |  |  |  |
| *nad4L* | 1132 | 1401 | 270 | 90 | ATG | TAA |  |  |  |
| *nad5* | 1401 | 4399 | 2999 | 662 | ATG | TAA | 2118 | 3130 |  |
| *cob* | 5028 | 6185 | 1158 | 386 | ATG | TAA |  |  |  |
| *trn*V* | 6568 | 6597 | 30 |  |  |  |  |  |  |
| *nad4* | 6933 | 8882 | 1950 | 658 | ATG | TAG |  |  |  |
| Orf2 | 8924 | 9892 | 969 | 323 | ATG | TAA |  |  |  |
| *nad1* | 10185 | 11300 | 1116 | 372 | ATG | TAA |  |  |  |
| *trn*C | 11663 | 11734 | 72 |  |  |  |  |  |  |
| *atp6* | 11875 | 12648 | 774 | 258 | ATG | TAA |  |  |  |
| *trn*M | 12863 | 12934 | 72 |  |  |  |  |  |  |
| *trn*H | 12981 | 13054 | 74 |  |  |  |  |  |  |
| *trn*Q | 13058 | 13129 | 72 |  |  |  |  |  |  |
| *trn*L | 13348 | 13430 | 83 |  |  |  |  |  |  |
| *trn*F | 13884 | 13956 | 73 |  |  |  |  |  |  |
| *trn*A | 14737 | 14808 | 72 |  |  |  |  |  |  |
| *trn*E | 14841 | 14913 | 73 |  |  |  |  |  |  |
| *trn*M | 15092 | 15164 | 73 |  |  |  |  |  |  |
| *trn*M | 15170 | 15240 | 71 |  |  |  |  |  |  |
| *trn*T | 15263 | 15333 | 71 |  |  |  |  |  |  |
| *trn*P | 18994 | 19066 | 73 |  |  |  |  |  |  |
| *trn*S | 19258 | 19342 | 85 |  |  |  |  |  |  |
| *trn*R | 19548 | 19619 | 72 |  |  |  |  |  |  |
| *trn*I | 19624 | 19695 | 72 |  |  |  |  |  |  |
| *trn*W | 19797 | 19868 | 72 |  |  |  |  |  |  |
| *trn*S | 20069 | 20148 | 80 |  |  |  |  |  |  |
| *trn*D | 20388 | 20459 | 72 |  |  |  |  |  |  |
| *trn*G | 20462 | 20534 | 73 |  |  |  |  |  |  |
| *trn*V | 21376 | 21448 | 73 |  |  |  |  |  |  |
| *nad6* | 21520 | 22107 | 588 | 196 | ATG | TAA |  |  |  |
| *trn*N | 22407 | 22477 | 71 |  |  |  |  |  |  |
| *cox2* | 22833 | 24697 | 1865 | 248 | TTA | TAG | 23355 | 24475 |  |
| *cox1* | 24704 | 27585 | 2882 | 539 | ATG | TTG | 25590 | 26854 |  |
| *trn*R | 28491 | 28561 | 71 |  |  |  |  |  |  |
| *trn*K | 30614 | 30696 | 83 |  |  |  |  |  |  |
| *trn*Y | 31266 | 31350 | 85 |  |  |  |  |  |  |
| *trn*N | 31446 | 31516 | 71 |  |  |  |  |  |  |
| *trn*V | 31582 | 31654 | 73 |  |  |  |  |  |  |
| *trn*K | 31684 | 31755 | 72 |  |  |  |  |  |  |
| *rps3* | 31769 | 33547 | 1779 | 593 | TTG | TAG |  |  |  |
| *cox3* | 34851 | 35660 | 810 | 270 | ATG | TAA |  |  |  |
| *nad2* | 35906 | 37693 | 1788 | 596 | ATG | TAA |  |  |  |
| *nad3* | 37694 | 38461 | 768 | 256 | ATG | TAA |  |  |  |
